# Environmental contamination during influenza A (H5N1) outbreaks in Cambodia

**DOI:** 10.1186/1753-6561-5-S1-P65

**Published:** 2011-01-10

**Authors:** Srey Viseth Horm, Ramona Alikiiteaga Gutiérrez, Sowath Ly, Sirenda Vong, Philippe Buchy

**Affiliations:** 1 Institut Pasteur du Cambodge, Phnom Penh, Cambodia

## 

In response to confirmed cases of H5N1 infection in humans or poultry, we conducted investigations in households of index case’s and in the surrounding vicinity. Environmental specimens such as mud, pond water, aquatic plants and animals, poultry carcasses, faeces, soil and dust were collected in 6 households from 3 Cambodian provinces between April 2007 and February 2010. Two techniques to concentrate influenza virus from water were used. The first was based on the biological property of the virus to agglutinate chicken red blood cells (RBCs). The second was based on an adsorption step on glass wool, followed by an elution step with a beef extract solution at alkaline pH, in combination with a second concentration step with poly-ethylene glycol (PEG). For mud samples, we used a method based on an elution step followed by a PEG-precipitation step. The hemagglutinin (HA), neuraminidase (NA) and matrix (MA) genes was amplified using a real-time RT-PCR (qRT-PCR) method. All samples that tested positive by qRT-PCR were inoculated into specific pathogen free (SPF) 9 to 11-day-old embryonated chicken eggs. From a total of 175 samples, 42 (24%) tested positive for H5N1 by qRT-PCR. Viral RNA was frequently detected in farm soil (66%), pond and puddle water (10%), mud (7%), live poultry’s cloacal or tracheal swabs (5%), feathers (2%), straw from poultry cages (5%), and poultry faeces (2%). The number of RNA copies was highest in the contaminated mud and straw collected during an outbreak in Takeo province in 2010 (about 4.5 x 10^5^ RNA copies per gram). Of the 42 positive specimens by qRT-PCR which were then inoculated in eggs, viable H5N1 could only be amplified from 3 samples. The longest persistence of viral RNA observed in the environmental specimens was 12 days following the last poultry death. Our study demonstrates that H5N1 RNA was frequently present on various environmental surfaces in the households of H5N1-infected patients and in the surrounding environment. We successfully detected viral RNA from mud and dry soil. However, the presence of RNA does not necessarily imply that the virus is still infectious or that human contamination could occur. We were only able to isolate viable virus from 3 out of 42 samples, and there was no correlation between these samples and those in which the highest quantity of RNA was detected. Nevertheless, the results underscore the potential role of the environment in H5N1 human and animal contamination as well as the importance for regular surveillance and disinfection of the surrounding environment following avian influenza outbreaks.

